# Systematic Review and Meta-Analysis of Proton Beam Therapy Versus Photon Radiotherapy for Medulloblastoma: TRP-Medulloblastoma 2025

**DOI:** 10.3390/cancers17132191

**Published:** 2025-06-29

**Authors:** Yinuo Li, Masashi Mizumoto, Yoshiko Oshiro, Kazushi Maruo, Masako Inaba, Takashi Saito, Sho Hosaka, Takashi Iizumi, Hiroko Fukushima, Ryoko Suzuki, Hazuki Nitta, Shosei Shimizu, Kei Nakai, Hideyuki Sakurai

**Affiliations:** 1 Department of Radiation Oncology, University of Tsukuba, Tsukuba 305-8576, Ibaraki, Japan; yli@pmrc.tsukuba.ac.jp (Y.L.); ooyoshiko@hotmail.com (Y.O.); saitoh@pmrc.tsukuba.ac.jp (T.S.); iizumi@pmrc.tsukuba.ac.jp (T.I.); hnitta@pmrc.tsukuba.ac.jp (H.N.); knakai@pmrc.tsukuba.ac.jp (K.N.); hsakurai@pmrc.tsukuba.ac.jp (H.S.); 2 Department of Radiation Oncology, Tsukuba Medical Center Hospital, Tsukuba 305-8558, Ibaraki, Japan; 3 Department of Biostatistics, Institute of Medicine, University of Tsukuba, Tsukuba 305-8576, Ibaraki, Japan; maruo@md.tsukuba.ac.jp; 4 Department of Pediatrics, University of Tsukuba Hospital, Tsukuba 305-8576, Ibaraki, Japan; minaba-tuk@md.tsukuba.ac.jp (M.I.); shohosaka@hotmail.com (S.H.); fkhiroko@md.tsukuba.ac.jp (H.F.); ryokosuzuki@md.tsukuba.ac.jp (R.S.); 5 Department of Child Health, Institute of Medicine, University of Tsukuba, Tsukuba 305-8576, Ibaraki, Japan; 6 Department of Pediatric Radiation Therapy Center, Hebei Yizhou Cancer Hospital, Zhuozhou 072750, China; 23s.shimizu@yz-proton.com

**Keywords:** meta-analysis, proton beam therapy, photon radiotherapy, pediatric medulloblastoma, survival outcomes

## Abstract

**Background/Objectives**: PBT enables precise targeting of tumors, which reduces side effects on surrounding organs, with particularly significant benefits for pediatric tumors. However, the efficacy of PBT compared to photon RT for pediatric medulloblastoma is unclear. A meta-analysis to assess the efficacy of PBT vs. photon RT for this disease was performed. **Methods**: Articles focusing on postoperative primary medulloblastoma from 1990 to 2022 were systematically reviewed and survival outcomes were extracted for medulloblastoma treated with PBT or photon RT. Meta-regression was used to identify predictive factors for treatment outcomes, including modality (PBT vs. photon RT), disease risk classification, gender, and age. **Results**: From 935 records, 18 articles were included (14 on photon RT, with 6 focusing on high-risk cases, and 4 on PBT). In standard-risk cases, the 1-, 3-, and 5-year overall survival (OS) rates were 95.5% (95% CI: 87.2–98.4%) vs. 96.7% (95% CI: 93.5–98.4%) (*p* = 0.1210); 89.8% (95% CI: 85.3–92.9%) vs. 88.0% (95% CI: 83.7–91.3%) (*p* = 0.5416); and 82.9% (95% CI: 76.6–87.6%) vs. 82.4% (95% CI: 77.2–86.5%) (*p* = 0.8313), respectively. For high-risk cases, the OS rates were 88.9% (95% CI: 85.5–91.6%); 73.6% (95% CI: 60.9–82.8%); and 68.6% (95% CI: 56.1–78.3%), respectively. The 1-, 3-, and 5-year progression-free survival (PFS) rates in standard-risk cases were 95.2% (95% CI: 91.8–97.2%) vs. 93.8% (95% CI: 89.4–96.5%) (*p* = 0.5275); 82.9% (95% CI: 77.3–87.2%) vs. 82.5% (95% CI: 80.1–84.7%) (*p* = 0.4294); and 79.6% (95% CI: 73.1–84.6%) vs. 77.0% (95% CI: 72.7–80.8%) (*p* = 0.3938), respectively. For high-risk cases, the PFS rates were 83.5% (95% CI: 77.0–88.3%); 64.8% (95% CI: 53.2–74.2%); and 60.4% (95% CI: 47.0–71.4%), respectively. Meta-regression analysis indicated no significant differences in 1- to 5-year OS and PFS between radiation modalities. Standard-risk cases were significantly associated with better OS and PFS. Younger age was significantly associated with 1- and 2-year OS and 1-year PFS, and male gender was significantly associated with 3-year OS and 3-year PFS. **Conclusions**: Postoperative irradiation for pediatric medulloblastoma using PBT has a therapeutic effect comparable to that of photon RT. This suggests that PBT is a useful option among RT modalities for medulloblastoma.

## Simple Summary

Medulloblastoma is one of the most common malignant brain tumors in children, and postoperative radiotherapy is essential for improving survival outcomes. Proton beam therapy (PBT) has gained attention due to its potential to reduce radiation exposure to healthy tissues, thereby minimizing long-term side effects in pediatric patients. However, whether PBT offers comparable efficacy to conventional photon radiotherapy (RT) remains unclear. In this systematic review and meta-analysis, we evaluated and compared survival outcomes in children with medulloblastoma treated with either PBT or photon RT. Our findings demonstrate that both modalities provide similar therapeutic efficacy in terms of overall and progression-free survival. Nonetheless, patients with high-risk disease continue to exhibit poorer outcomes regardless of the radiation modality used. These results suggest that PBT is a viable treatment option for pediatric medulloblastoma, particularly in light of its potential to reduce long-term toxicity.

## 1. Introduction

Medulloblastoma is a common malignant pediatric brain tumor that accounts for about 20% of central nervous system tumors in children [1]. Typically arising in the posterior fossa, it poses unique treatment challenges due to its tendency to disseminate through the cerebrospinal fluid, which complicates disease management [2]. Standard management includes surgical resection, radiotherapy (RT), and chemotherapy, with the aim to maximize tumor control and minimize recurrence [3]. However, despite advances in these modalities, achieving optimal outcomes with minimal long-term side effects remains challenging.

Radiation therapy is essential in medulloblastoma treatment for eliminating residual disease post surgery [4]. Patients typically receive craniospinal irradiation (CSI) with a boost dose to the tumor bed [5]. While effective, photon RT can expose surrounding healthy tissues to radiation, and this may lead to long-term side effects, especially in pediatric patients [6]. Proton beam therapy (PBT) has emerged as an alternative that offers precise radiation delivery that spares adjacent normal tissues [7]. This precision is particularly advantageous for children, since it potentially reduces the risk of cognitive and developmental impairments associated with radiation exposure [8,9].

Due to the relative rarity of pediatric medulloblastoma and the limited availability of PBT facilities, robust comparative data between PBT and photon RT are scarce. To address this gap, the Tsukuba Review Project (TRP) initiated a systematic review and meta-analysis to evaluate the efficacy of PBT vs. photon RT in treating medulloblastoma [10,11,12]. The aim of this study is to provide insights into survival outcomes and inform clinical decision-making for pediatric patients with medulloblastoma.

## 2. Materials and Methods

### 2.1. Selection Criteria for Meta-Analysis

**Figure 1:**
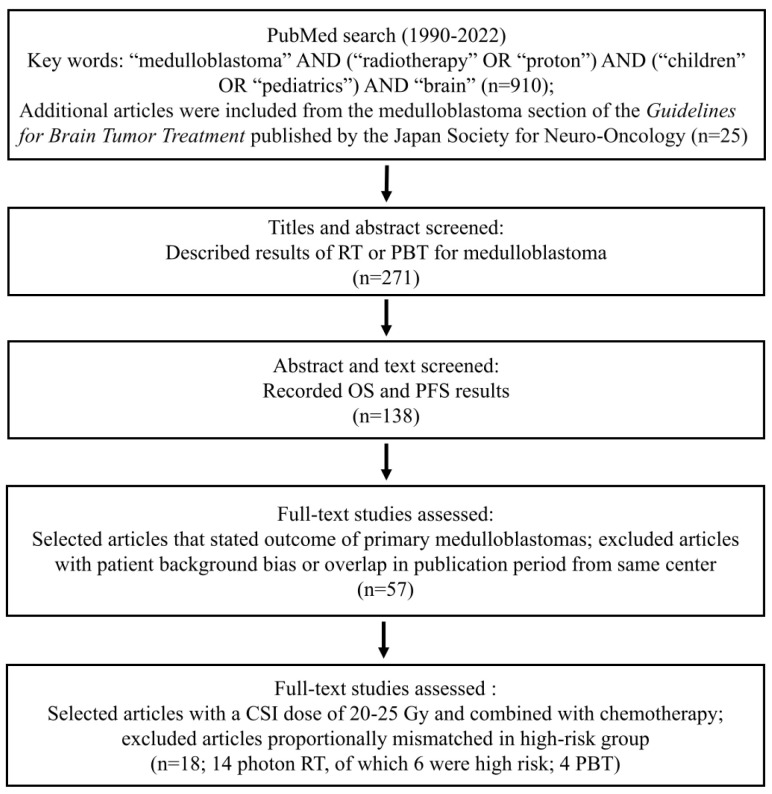
Flowchart of study selection. The flowchart illustrates the systematic selection of studies for the meta-analysis. RT: radiotherapy; PBT: proton beam therapy; OS: overall survival; PFS: progression-free survival; CSI: craniospinal irradiation.

The review was conducted in compliance with the Preferred Reporting Item for Systematic Reviews and Meta-Analyses (PRISMA) guidelines and recommendations [13]. Only English-language articles were selected. All retrieved articles were screened by more than two reviewers. The selection process followed a rigorous screening protocol to ensure that only studies meeting specific inclusion criteria were analyzed. This process is summarized in Figure 1. Although this review was conducted in 2025, the included studies reported data collected between 1990 and 2022. An initial PubMed search was conducted for publications from 1990 to 2022 using the keywords “medulloblastoma” AND (“radiotherapy” OR “proton”) AND (“children” OR “pediatrics”) AND “brain,” yielding 910 articles. In addition, 25 articles from the medulloblastoma section of the Guidelines for Brain Tumor Treatment published by The Japan Society for Neuro-Oncology were also selected [14]. The titles and abstracts of these studies were screened to identify those specifically describing treatment outcomes of photon RT or PBT for medulloblastoma, narrowing the pool to 271 articles. Further screening at the abstract and full-text levels focused on studies that reported both overall survival (OS) and progression-free survival (PFS), reducing the number to 138 articles. Full-text assessments then selected studies reporting outcomes for primary medulloblastoma, excluding those with patient background bias or publication complete overlap from the same centers, resulting in 57 articles. Only studies using a CSI dose of 20–25 Gy combined with chemotherapy were selected, while those with proportional mismatches in high-risk groups were excluded, yielding a final selection of 18 studies (14 photon RT, of which 6 were exclusively high-risk cases, and 4 PBT). Original data collected from each article included authors, year of publication, study design, number of patients, age, sex, treatment modality (photon RT vs. PBT), standard-risk percentage, high-risk percentage, chemotherapy rate, radiation dose, 1- to 5-year OS and 1- to 5-year PFS. Information on the extent of resection and molecular subgroup classification was not available in all studies. If OS and PFS were not specified in the main text, they were estimated from figures.

### 2.2. Statistical Analysis

Random effects meta-analyses of 1- to 5-year OS and PFS were performed for each modality and forest plots were drawn. For studies with missing accuracy data, missing values were imputed using the number of cases, risk set size at each year, and mean dropout rate. Heterogeneity in each meta-analysis was evaluated by I-square statistics. Random-effects meta-regression with modality as an explanatory variable was performed for each outcome to compare the modalities. All analyses were performed using R (R Core Team, Vienna, Austria) and its accompanying meta package [15].

## 3. Results

A total of 18 articles met the inclusion criteria [16,17,18,19,20,21,22,23,24,25,26,27,28,29,30,31,32]. Among these, 14 focused on photon RT, of which 6 exclusively studied high-risk populations, and 4 focused on PBT. Forest plots were generated for three groups: a PBT group with mostly standard-risk cases (four studies), a photon RT group with mostly standard-risk cases (eight studies), and a photon RT group with high-risk cases (six studies). The selected articles and some details of these articles are listed in Table 1.

**Figure 2:**
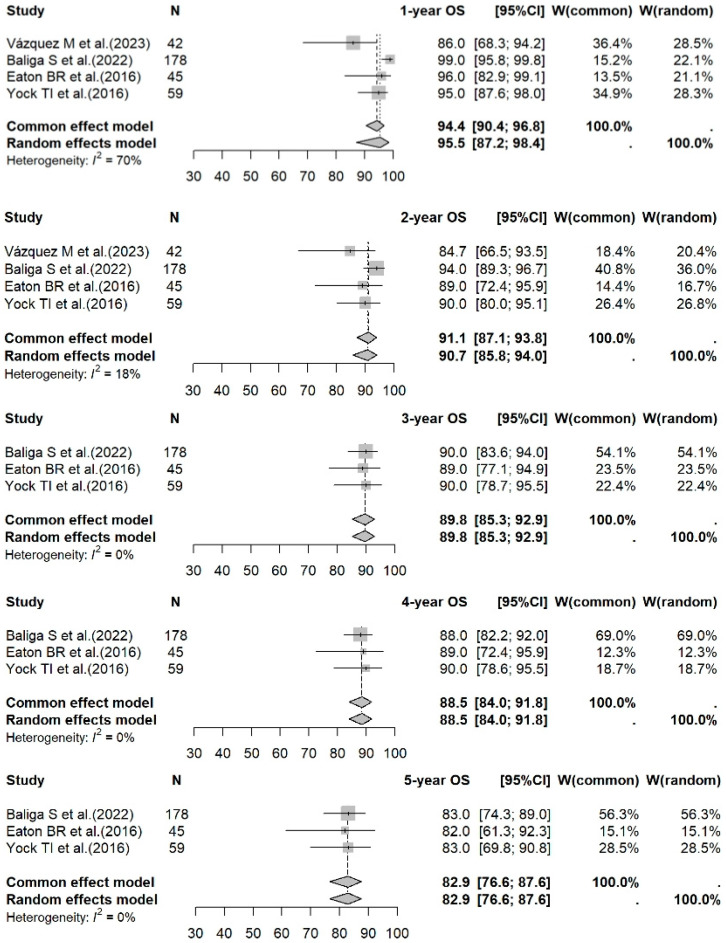
Forest plot of 1- to 5-year OS rates for PBT. The forest plot displays the 1- to 5-year OS rates for studies on PBT for medulloblastoma. Each data point represents an individual study—Vázquez M et al. [16], Baliga S et al. [17], Eaton BR et al. [18], and Yock TI et al. [19]—with the corresponding CI shown as horizontal lines. The pooled estimates of OS rates are displayed, providing a summary of the survival outcomes associated with PBT across different time points. OS: overall survival; PBT: proton beam therapy; CI: confidence interval.

**Figure 3:**
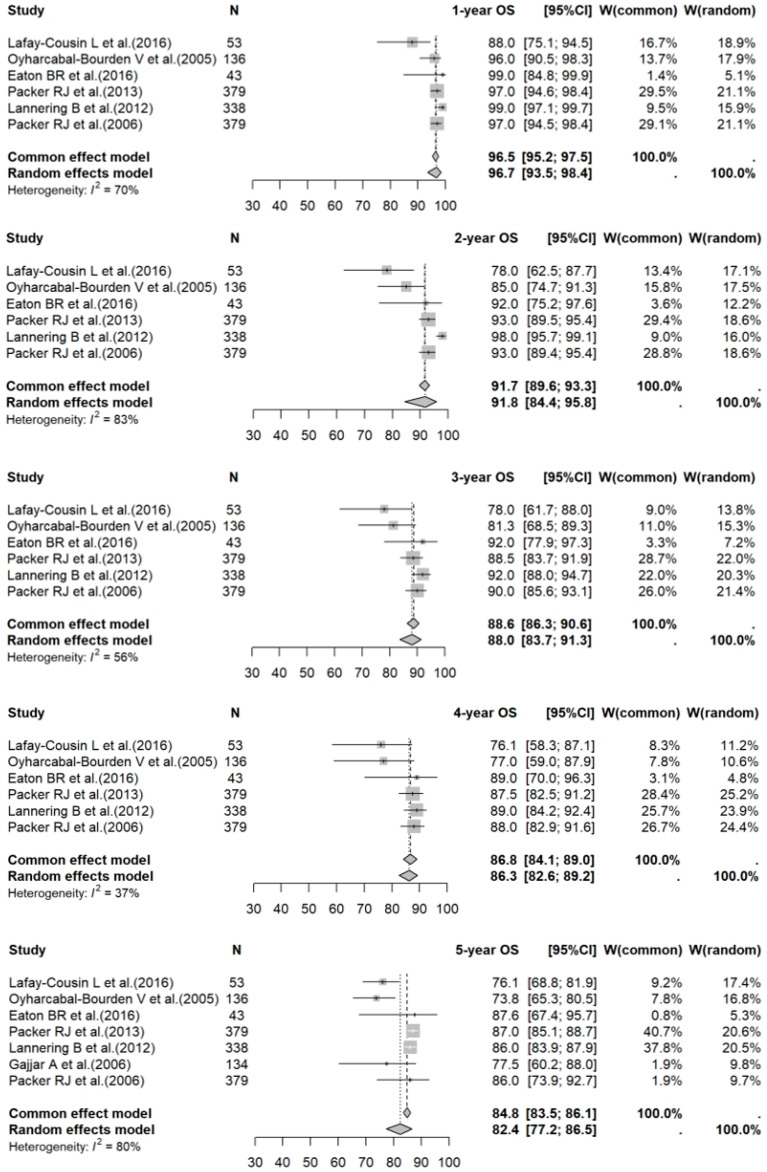
Forest plot of 1- to 5-year OS rates for photon RT. The forest plot illustrates the 1- to 5-year OS rates for studies on photon RT for medulloblastoma. Each data point represents an individual study—Lafay-Cousin L et al. [21], Oyharcabal-Bourden V et al. [22], Eaton BR et al. [18], Packer RJ et al. [23], Lannering B et al. [24], Gajjar A et al. [25], and Packer RJ et al. [26]—with the corresponding CI depicted as horizontal lines. The pooled estimates of OS rates provide a summary of survival outcomes associated with photon RT across different time points. OS: overall survival; RT: radiotherapy; CI: confidence interval.

**Figure 4:**
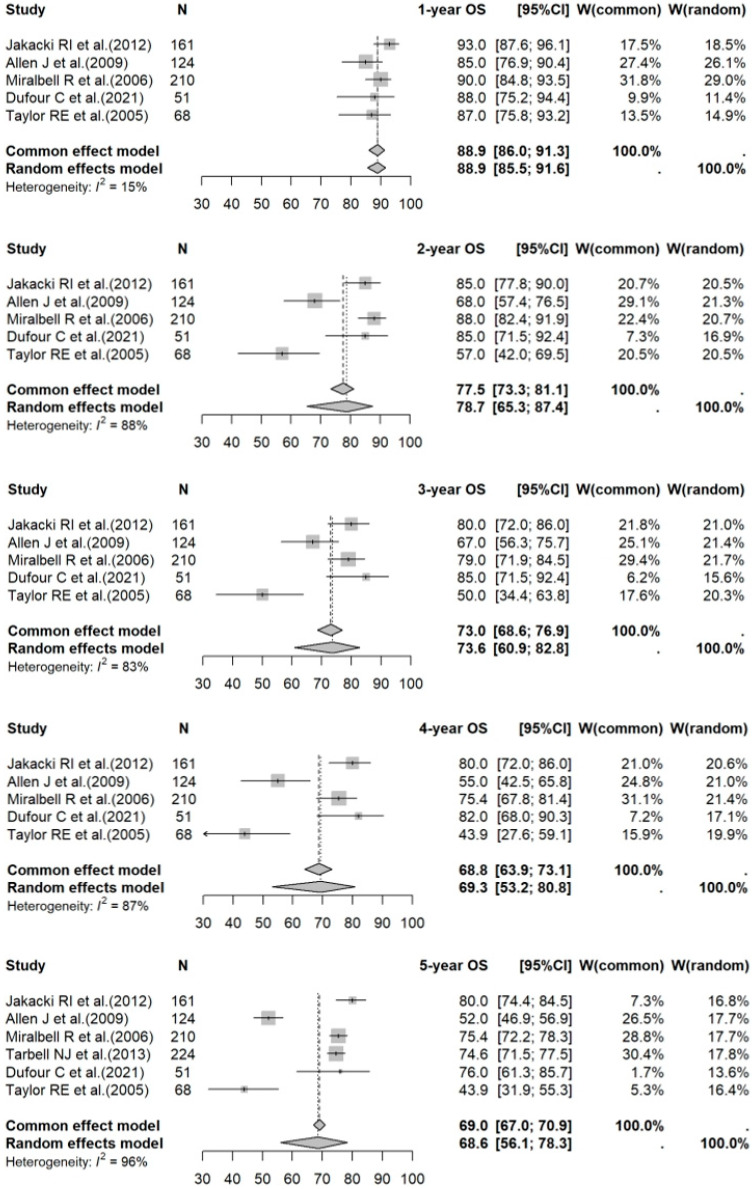
Forest plot of 1- to 5-year OS rates for photon RT in high-risk cases. The forest plot displays the 1- to 5-year OS rates for studies on photon RT in high-risk medulloblastoma populations. Each data point represents an individual study—Jakacki RI et al. [27], Allen J et al. [28], Miralbell R et al. [29], Tarbell NJ et al. [30], Dufour C et al. [31], and Taylor RE et al. [32]—with the corresponding CI shown as horizontal lines. The pooled estimates provide a summary of survival outcomes for high-risk patients receiving photon RT over different time periods. OS: overall survival; RT: radiotherapy; CI: confidence interval.

A meta-analysis of data for PBT vs. photon RT in standard-risk cases gave 1- to 5-year OS rates of 95.5% (95% CI: 87.2–98.4%) vs. 96.7% (95% CI: 93.5–98.4%) (*p* = 0.1210); 90.7% (95% CI: 85.8–94.0%) vs. 91.8% (95% CI: 84.4–95.8%) (*p* = 0.4909); 89.8% (95% CI: 85.3–92.9%) vs. 88.0% (95% CI: 83.7–91.3%) (*p* = 0.5416); 88.5% (95% CI: 84.0–91.8%) vs. 86.3% (95% CI: 82.6–89.2%) (*p* = 0.4170); and 82.9% (95% CI: 76.6–87.6%) vs. 82.4% (95% CI: 77.2–86.5%) (*p* = 0.8313), respectively. For the high-risk photon RT cases, the 1- to 5-year OS rates were 88.9% (95% CI: 85.5–91.6%); 78.7% (95% CI: 65.3–87.4%); 73.6% (95% CI: 60.9–82.8%); 69.3% (95% CI: 53.2–80.8%); and 68.6% (95% CI: 56.1–78.3%), respectively. Forest plots for OS across the three groups are shown in Figure 2, Figure 3 and Figure 4.

**Figure 5:**
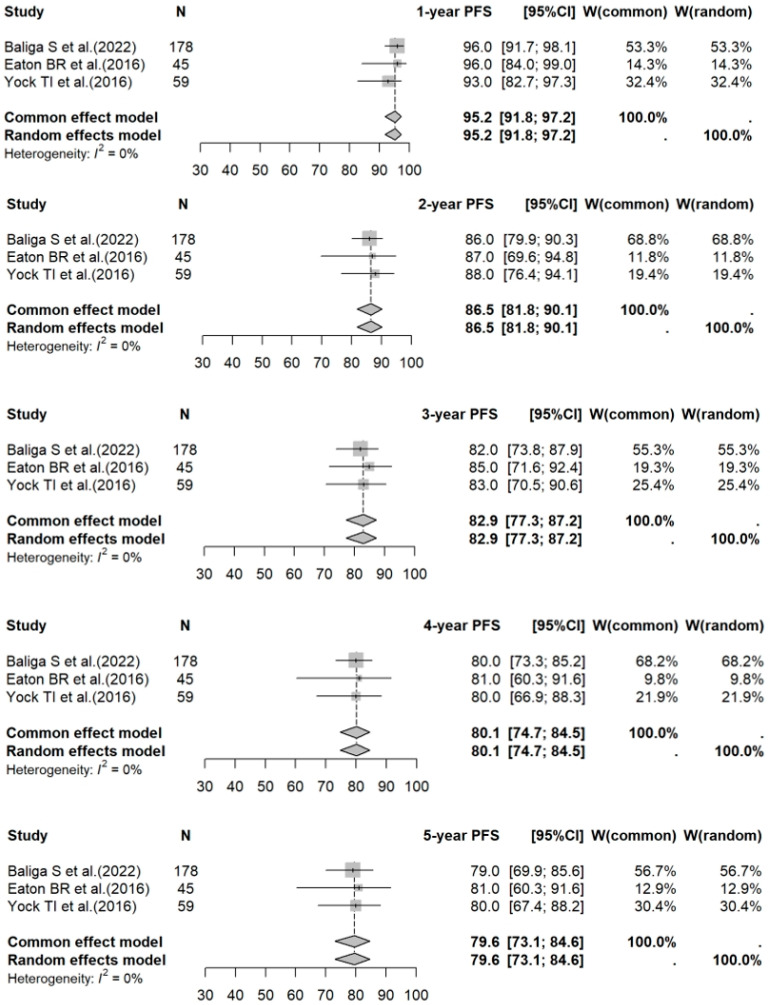
Forest plot of 1- to 5-year PFS rates for PBT. The forest plot displays the 1- to 5-year PFS rates for studies on PBT for medulloblastoma. Each data point represents an individual study—Baliga S et al. [17], Eaton BR et al. [18], and Yock TI et al. [19]—with horizontal lines indicating the corresponding CI. The pooled estimates provide a summary of PFS outcomes associated with PBT over different time intervals. PFS: progression-free survival; PBT: proton beam therapy; CI: confidence interval.

**Figure 6:**
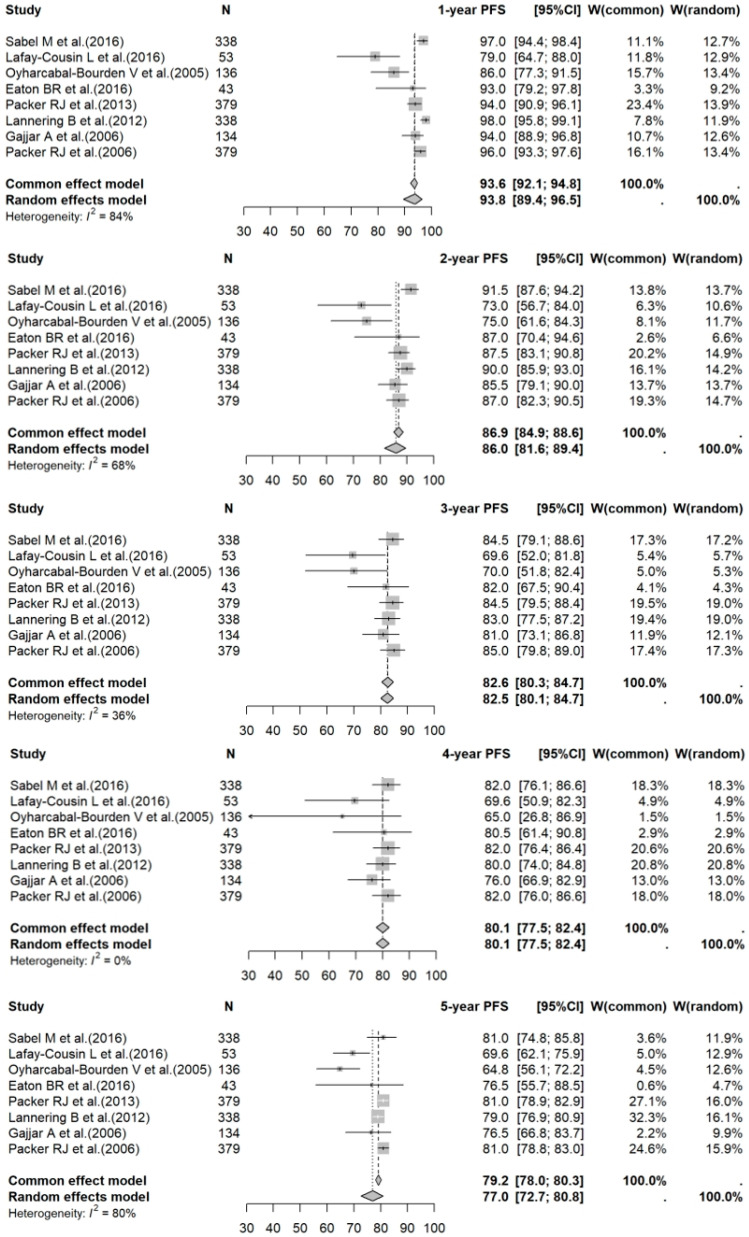
Forest plot of 1- to 5-year PFS rates for photon RT. The forest plot illustrates the 1- to 5-year PFS rates for studies on photon RT for medulloblastoma. Each data point represents an individual study—Sabel M et al. [20], Lafay-Cousin L et al. [21], Oyharcabal-Bourden V et al. [22], Eaton BR et al. [18], Packer RJ et al. [23], Lannering B et al. [24], Gajjar A et al. [25], and Packer RJ et al. [26]—with the corresponding CI depicted as horizontal lines. The pooled estimates of PFS rates provide a summary of PFS outcomes associated with photon RT across different time points. PFS: progression-free survival; RT: radiotherapy; CI: confidence interval.

**Figure 7:**
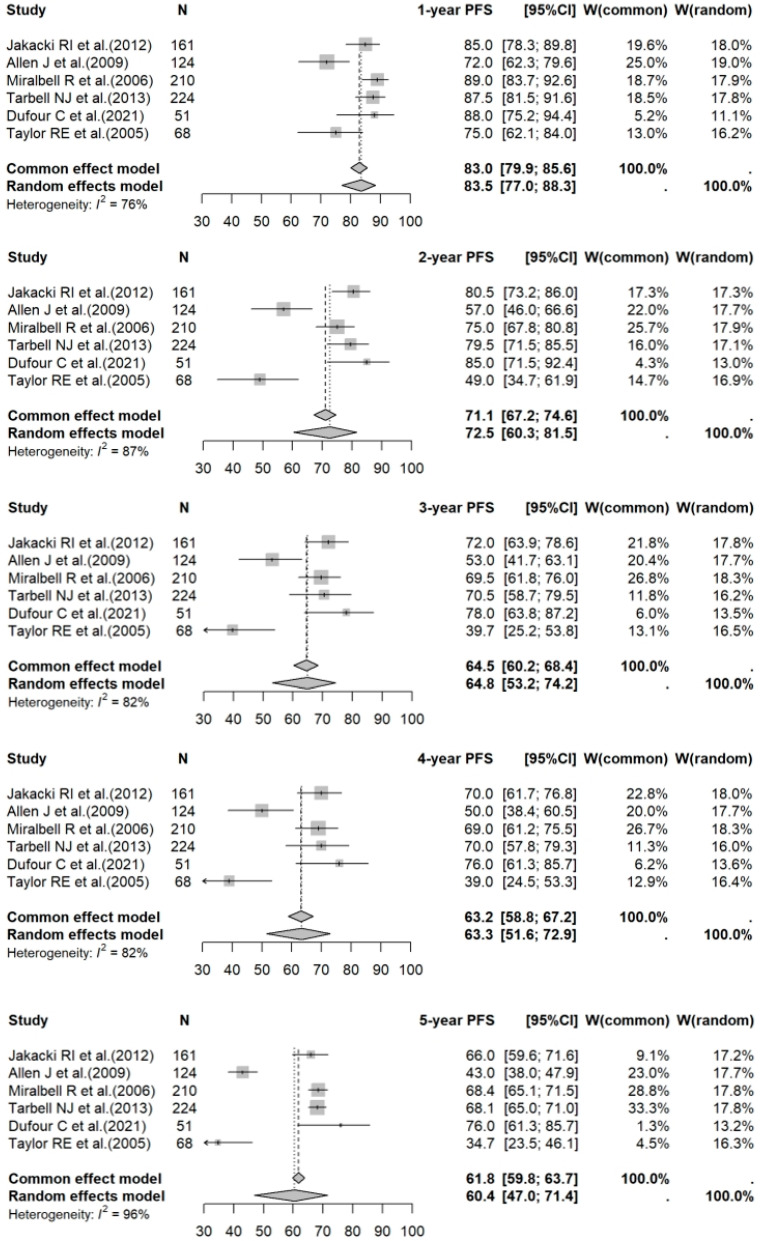
Forest plot of 1- to 5-year PFS rates for photon RT in high-risk cases. The forest plot illustrates the 1- to 5-year PFS rates for studies on photon RT in high-risk medulloblastoma populations. Each data point represents an individual study—Jakacki RI et al. [27], Allen J et al. [28], Miralbell R et al. [29], Tarbell NJ et al. [30], Dufour C et al. [31], and Taylor RE et al. [32]—with the corresponding CI shown as horizontal lines. The pooled estimates provide a summary of PFS outcomes for high-risk cases receiving photon RT over different time periods. PFS: progression-free survival; RT: radiotherapy; CI: confidence interval.

The 1- to 5-year PFS rates for PBT vs. photon RT in standard-risk cases were 95.2% (95% CI: 91.8–97.2%) vs. 93.8% (95% CI: 89.4–96.5%) (*p* = 0.5275); 86.5% (95% CI: 81.8–90.1%) vs. 86.0% (95% CI: 81.6–89.4%) (*p* = 0.5172); 82.9% (95% CI: 77.3–87.2%) vs. 82.5% (95% CI: 80.1–84.7%) (*p* = 0.4294); 80.1% (95% CI: 74.7–84.5%) vs. 80.1% (95% CI: 77.5–82.4%) (*p* = 0.5810); and 79.6% (95% CI: 73.1–84.6%) vs. 77.0% (95% CI: 72.7–80.8%) (*p* = 0.3938), respectively. For high-risk photon RT cases, the 1- to 5-year PFS rates were: 83.5% (95% CI: 77.0–88.3%); 72.5% (95% CI: 60.3–81.5%); 64.8% (95% CI: 53.2–74.2%); 63.3% (95% CI: 51.6–72.9%); and 60.4% (95% CI: 47.0–71.4%), respectively. Forest plots for PFS across the three groups are shown in Figure 5, Figure 6 and Figure 7.

Meta-regression analyses of 1- to 5-year OS and 1- to 5-year PFS were performed using potential predictive factors for modality (photon RT vs. PBT), risk classification (standard vs. high), male ratio (Male), and age. The extent of surgery and molecular subgroup were poorly documented and therefore not included in the meta-regression analysis. The results are shown in Table 2. The risk status emerged as a consistent predictor of outcomes and standard risk showed a significant association with better OS at all time points, with the effect being particularly strong for 3-year OS (*p* < 0.0001). A standard-risk status was also significantly associated with better PFS across all years and was also highest for 3-year PFS (*p* = 0.0009). In addition, age showed a negative correlation with 1-year OS (*p* = 0.0021), 2-year OS (*p* = 0.0453), and 1-year PFS (*p* = 0.0458), suggesting that younger patients had better short-term outcomes. Gender played a role in mid-term outcomes, with higher values for Male positively influencing 3-year OS (*p* = 0.0142) and 3-year PFS (*p* = 0.0431). No statistically significant differences were found between PBT and photon RT for effect on OS or PFS, indicating that these modalities have similar efficacy in treatment of patients with medulloblastoma.

## 4. Discussion

This systematic review and meta-analysis compared the therapeutic effects of PBT and photon RT in pediatric medulloblastoma, with a focus on OS and PFS over different time intervals. Additionally, a subgroup analysis was conducted based on different risk groups. Notably, the findings suggest that PBT and photon RT have similar therapeutic efficacy for pediatric medulloblastoma, with no significant difference in OS or PFS over a 1- to 5-year follow-up period. However, risk factor analysis revealed that studies involving a higher proportion of high-risk cases had poorer OS and PFS outcomes, regardless of whether PBT or photon RT was used. This effect was particularly pronounced in studies in which high-risk cases accounted for 100% of the cohort, which showed a significant decline in both OS and PFS.

Although the therapeutic efficacy of PBT is similar to that of photon RT, PBT has distinct advantages, particularly for pediatric patients [33]. This is because PBT minimizes radiation exposure to surrounding healthy tissues, which is crucial for reducing long-term side effects such as cognitive impairment, endocrine dysfunction, and secondary malignancies, all of which have a more significant impact on children than adults [34]. In 2019, a meta-analysis including 12 studies showed that PBT offered a dosimetric advantage over 3D conformal RT for target homogeneity and had significantly better outcomes in sparing organs at risk (OARs), such as the brainstem, normal brain, and hippocampus, which supported the superiority of PBT over photon RT in terms of dose distribution, especially in sparing doses to normal developing tissues [35]. Similarly, a comparative cohort study showed that PBT has equivalent therapeutic efficacy with better toxicity control compared to photon RT. Patients treated with PBT had superior neurocognitive outcomes, a lower incidence of hypothyroidism (23% vs. 69%), reduced acute toxicity (including bone marrow suppression, esophagitis, diarrhea, weight loss, and nausea/vomiting), and a lower 10-year cumulative incidence of secondary malignancies (2.1–4.9% vs. 8%). The 10-year OS and PFS rates were comparable to those reported with photon RT [36]. Moreover, a retrospective analysis of 97 cases of pediatric medulloblastoma showed no difference in OS between treatment with PBT-based CSI and photon RT; however, hematological toxicity was significantly lower with PBT [37]. These findings align with those in the current study, and emphasize that while PBT and photon RT have similar efficacy in treating pediatric medulloblastoma, PBT is a vital treatment option, particularly for protecting healthy tissues in children, in whom tissue preservation is critical. However, it is important to note that the availability of PBT remains limited in many regions due to the high cost of treatment and the limited number of specialized facilities, which may influence treatment selection and access in real-world clinical settings. Given these limitations, photon radiotherapy continues to play a crucial role in the treatment of pediatric medulloblastoma and remains an essential component of current clinical practice.

The current analysis identified risk status as a significant factor in survival outcomes, with high-risk patients showing poorer OS and PFS across all time points. This aligns with clinical expectations, as high-risk medulloblastoma often presents with more aggressive disease characteristics, higher tumor burden, or metastatic spread, making treatment more challenging and recurrence more likely [38]. Sirachainan et al. found that patients with high-risk medulloblastoma have significantly lower OS and PFS compared to standard-risk cases, with average 5-year PFS and OS of 55% and 65%, respectively [39]. In a study of postoperative RT combined with chemotherapy for medulloblastoma, the 5-year PFS decreased from 62.9 ± 10% for average-risk cases to 48.9 ± 13% for high-risk cases, and the 5-year OS decreased from 70.4 ± 9.5% to 49.7 ± 13% in these groups [40]. These previous results align with our findings; thus, whether using PBT or photon RT, OS and PFS for high-risk cases are poor. In such cases, even the precision offered by PBT might be insufficient to counteract the aggressive nature of the disease, which highlights the need for additional treatment strategies beyond RT alone to improve outcomes for these patients [41].

Another important consideration is the heterogeneity within medulloblastoma and among other pediatric neuroepithelial tumors [42]. Certain entities—such as atypical teratoid/rhabdoid tumor (AT/RT) and supratentorial primitive neuroectodermal tumors (sPNET)—may exhibit overlapping cellular characteristics with medulloblastoma, making accurate diagnosis challenging in some cases [43]. Factors such as tumor location (supratentorial vs. infratentorial), age at diagnosis, and the presence of molecular markers (e.g., FOXR2, BCOR, CIC, MN1 alterations) are important for differential diagnosis but are not definitive in isolation. While the present study focused on clinically diagnosed medulloblastoma, the majority of included studies were retrospective and based on clinical diagnoses without detailed molecular characterization. As a result, the possibility of misclassification or inclusion of biologically distinct tumor types cannot be completely excluded. Future meta-analyses should incorporate molecular diagnostic data where available to enable more accurate and biologically meaningful comparisons of treatment outcomes.

In this study, age was identified as a predictive factor, with younger patients having significantly better 1- and 2-year OS and 1-year PFS. It is well-established that children under the age of 3 with medulloblastoma are generally considered to be high-risk due to their developing brains, which often precludes use of RT, and treatment primarily relies on high-dose chemotherapy [44]. However, in this meta-analysis, only one of the 18 included studies had a median patient age below 3 years, with most involving patients older than 3 years. Thus, our findings indicate that among patients who are mostly older than 3 years, younger patients have better 1-year OS (*p* = 0.0021), 2-year OS (*p* = 0.0453), and 1-year PFS (*p* = 0.0458). This finding underscores the importance of personalizing treatment regimens to optimize outcomes for different age groups. Gender also had a slight impact on mid-term survival outcomes, with a higher proportion of male patients associated with better 3-year OS and PFS. The underlying reasons for this observation remain unclear, but it is possible that physiological or hormonal differences between genders may influence the response of medulloblastoma to treatment or the ability of the patient to tolerate therapy.

One limitation of the current study is the scarcity of large-volume comparative studies, particularly for PBT, due to its limited availability and the high cost of treatment. Consequently, large-scale, multicenter studies or registries could play a crucial role in collecting more robust data, particularly for high-risk groups and other subpopulations with distinct clinical needs. Another limitation is the reliance on OS and PFS as the primary endpoints, which, while important, do not capture the full scope of quality of life, long-term functional outcomes, or incidence of late-onset side effects, especially in pediatric cases. Future studies should consider incorporating these additional parameters to better capture the impact of PBT on overall patient well-being. Furthermore, it should be noted that some of the included proton therapy studies may involve some overlapping patients, as several were conducted as multi-institutional studies. However, due to the limited number of available studies on proton therapy for medulloblastoma and the fact that the overlaps are only partial—with each study contributing unique patient subsets, these studies were included for analysis. Nevertheless, this limitation is acknowledged, and future meta-analyses—once more independent data become available—should consider excluding overlapping datasets to improve the validity of the conclusions.

Another methodological consideration is the potential impact of rounding inaccuracy on the pooled effect estimates. Most of the included studies reported hazard ratios, survival rates, or other outcomes using one or two decimal places, which may introduce minor rounding errors. Since the meta-analysis was conducted based on published aggregate data, the cumulative effect of such rounding could not be fully quantified. However, all analyses were performed using the most precise values available from each source, and the rounding margins are considered small. Therefore, the influence of rounding inaccuracy on the overall results is expected to be minimal. Nonetheless, this limitation should be acknowledged.

The TRP is currently undertaking a series of systematic reviews and meta-analyses to compare the therapeutic efficacy of PBT and photon RT across various representative pediatric tumors [10,11,12] with the aim of confirming the value of PBT in pediatric cases. A prospective registry study is also ongoing in Japan to evaluate long-term adverse events, such as secondary cancers.

## 5. Conclusions

PBT and photon RT provide similar survival benefits as postoperative treatment for medulloblastoma. However, the precision and tissue-sparing advantages of PBT makes it an important option for pediatric medulloblastoma. High-risk patients continue to face challenges in achieving long-term survival, even with different radiation modalities. Further research is also needed to evaluate long-term quality of life, functional outcomes, and the incidence of late-onset side effects, particularly in pediatric cases.

## Figures and Tables

**Table 1:** List of selected articles. P: Prospective Study. R: Retrospective Study. OS: Overall Survival.

Author	Reference	Year	Modality	Study Design	n	Male (%)	Median Age (Year)	Standard Risk (%)	High Risk (%)	3y OS (%)	5y OS (%)
Vázquez M	[16]	2023	Proton	R	42	67.6	7.4	-	-	-	-
Baliga S	[17]	2022	Proton	R	178	57.9	8.1	57.3	33.7	90	83
Eaton BR	[18]	2016	Proton	R	45	55.6	6.2	100	0	89	82
Yock TI	[19]	2016	Proton	P	59	56	6.6	66	24	90	83
Sabel M	[20]	2016	Photon	P	338	-		100	0	-	-
Lafay-Cousin L	[21]	2016	Photon	R	53	56.6	2	-	-	78	76.1
Oyharcabal-Bourden V	[22]	2005	Photon	P	136	58.8	8	100	0	81.3	73.8
Eaton BR	[18]	2016	Photon	R	43	67.4	8.2	100	0	92	87.6
Packer RJ	[23]	2013	Photon	P	379	58.8	6.8	-	-	88.5	87
Lannering B	[24]	2012	Photon	P	338	62.4	8	100	0	92	86
Gajjar A	[25]	2006	Photon	P	134	67.2	7.6	-	35.8	-	77.5
Packer RJ	[26]	2006	Photon	P	379	59	7	-	-	90	86
Jakacki RI	[27]	2012	Photon	P	161	58	8.7	0	100	80	80
Allen J	[28]	2009	Photon	P	124	64.5	7.8	0	100	67	52
Miralbell R	[29]	2006	Photon	P	210	-	-	0	100	79	75.4
Tarbell NJ	[30]	2013	Photon	P	224	59	7.8	0	100	-	74.6
Dufour C	[31]	2021	Photon	P	51	66.7	8	0	100	85	76
Taylor RE	[32]	2005	Photon	P	68	29	7.8	0	100	50	43.9

**Table 2:** Meta-regression analysis of potential predictive factors for 1- to 5-year OS and 1- to 5-year PFS. OS: Overall Survival. PFS: Progression-Free Survival. SE: Standard Error. Z: Z-value. *p*: *p*-value. CI.lb: Lower Bound of Confidence Interval. CI.ub: Upper Bound of Confidence Interval. Male: Male ratio.

Factors	Estimate	SE	Z	*p*	CI.lb	CI.ub
1-year OS						
Modality	−0.6449	0.4159	−1.5506	0.1210	−1.4600	0.1703
Risk classification	1.1791	0.4353	2.7090	0.0067	0.3260	2.0322 **
Male	0.0071	0.0156	0.4558	0.6485	−0.0234	0.0376
Age	−0.3232	0.1052	−3.0733	0.0021	−0.5293	−0.1171 **
2-year OS						
Modality	−0.2810	0.4079	−0.6889	0.4909	−1.0805	0.5185
Risk classification	1.1422	0.4470	2.5551	0.0106	0.2660	2.0183 *
Male	−0.0185	0.0174	−1.0651	0.2868	−0.0527	0.0156
Age	−0.2219	0.1108	−2.0021	0.0453	−0.4391	−0.0047 *
3-year OS						
Modality	0.1749	0.2866	0.6103	0.5416	−0.3868	0.7367
Risk classification	1.1784	0.3023	3.8974	<0.0001	0.5858	1.7710 ***
Male	−0.0264	0.0108	−2.4519	0.0142	−0.0475	−0.0053 *
Age	−0.1193	0.0742	−1.6075	0.1079	−0.2648	0.0262
4-year OS						
Modality	0.2840	0.3500	0.8117	0.4170	−0.4019	0.9699
Risk classification	1.2310	0.3671	3.3531	0.0008	0.5115	1.9506 ***
Male	−0.0246	0.0138	−1.7876	0.0738	−0.0517	0.0024
Age	−0.0929	0.0899	−1.0335	0.3014	−0.2691	0.0833
5-year OS						
Modality	0.0749	0.3518	0.2130	0.8313	−0.6146	0.7644
Risk classification	0.7725	0.3596	2.1482	0.0317	0.0677	1.4772 *
Male	−0.0202	0.0138	−1.4678	0.1422	−0.0471	0.0068
Age	−0.0527	0.0795	−0.6630	0.5073	−0.2086	0.1031
1-year PFS						
Modality	0.3010	0.4764	0.6318	0.5275	−0.6327	1.2346
Risk classification	1.5409	0.4882	3.1561	0.0016	0.5840	2.4979 **
Male	−0.0109	0.0178	−0.6105	0.5415	−0.0457	0.0240
Age	−0.2165	0.1084	−1.9977	0.0458	−0.4290	−0.0041 *
2-year PFS						
Modality	0.2193	0.3385	0.6477	0.5172	−0.4442	0.8828
Risk classification	0.8931	0.3520	2.5373	0.0112	0.2032	1.5830 *
Male	−0.0240	0.0135	−1.7790	0.0752	−0.0505	0.0024
Age	−0.0919	0.0857	−1.0727	0.2834	−0.2599	0.0760
3-year PFS						
Modality	0.2110	0.2669	0.7903	0.4294	−0.3122	0.7341
Risk classification	0.9218	0.2787	3.3081	0.0009	0.3757	1.4680 ***
Male	−0.0216	0.0107	−2.0229	0.0431	−0.0426	−0.0007 *
Age	−0.0579	0.0711	−0.8147	0.4153	−0.1972	0.0814
4-year PFS						
Modality	0.1416	0.2566	0.5520	0.5810	−0.3613	0.6446
Risk classification	0.7932	0.2623	3.0238	0.0025	0.2791	1.3074 **
Male	−0.0189	0.0103	−1.8372	0.0662	−0.0392	0.0013
Age	−0.0487	0.0717	−0.6792	0.4970	−0.1891	0.0918
5-year PFS						
Modality	0.2601	0.3050	0.8527	0.3938	−0.3377	0.8578
Risk classification	0.8671	0.3169	2.7363	0.0062	0.2460	1.4882 **
Male	−0.0211	0.0121	−1.7488	0.0803	−0.0448	0.0025
Age	−0.0223	0.0687	−0.3248	0.7454	−0.1569	0.1123

## Data Availability

The datasets used and/or analyzed during the current study are available from the corresponding author on reasonable request.

## Abbreviations

The following abbreviations are used in this manuscript:

PBT	Proton Beam Therapy
RT	Radiotherapy
TRP	Tsukuba Review Project
OS	Overall Survival
PFS	Progression-Free Survival
CSI	Craniospinal Irradiation
OAR	Organ at Risk
PRISMA	Preferred Reporting Item for Systematic Reviews and Meta-Analyses
SE	Standard Error
AT/RT	Atypical Teratoid/Rhabdoid Tumor
sPNET	Supratentorial Primitive Neuroectodermal Tumor
